# Supplementary material for: Insights into metabolic heterogeneity of colorectal cancer gained from fluorescence lifetime imaging
Source: eLife. 2024 Aug 28;13:RP94438. doi: 10.7554/eLife.94438 (PMC11357354; doi:10.7554/eLife.94438)
Supplement: Supplementary file 1. [file elife-94438-supp1.docx]

| **Cell lines in vitro** | | | |
| --- | --- | --- | --- |
|  | HT29 | HCT116 | CaCo2 |
| HCT116 | 8.56˟10^-108^ | - | - |
| CaCo2 | 1.58˟10^-71^ | 1.72˟10^-83^ | - |
| CT26 | 2.53˟10^-166^ | 2.09˟10^-36^ | 2.45˟10^-92^ |
| **Tumors in vivo** | | | |
|  | HT29 | HCT116 | CaCo2 |
| HCT116 | 0.1 | - | - |
| CaCo2 | 0.057 | 0.057 | - |
| CT26 | 0.727 | 0.064 | 0.0028 |
